# Citric Acid–Urea Modification of Dual-Plasticized Starch Adhesives for Medium-Density Fiberboard Manufacturing

**DOI:** 10.3390/polym18192400

**Published:** 2026-10-01

**Authors:** Meysam Mehdinia, Saeed Kamrani, Peyman Ahmadi, Sara Nabipoor, Sogand Ghafari Movahed, Petar Antov, Ali Dorieh, Viktor Savov, Stoyko Petrin, Lee Seng Hua

**Affiliations:** 1 Wood and Forest Products Science Research Division, Research Institute of Forests and Rangelands, Agricultural Research, Education and Extension Organization (AREEO), Tehran 14968-13111, Iran; meysammehdinia@gmail.com; 2 Department of Wood and Paper Science and Technology, Faculty of Natural Resources, University College of Agricultural & Natural Resources, University of Tehran, Karaj 77871-31587, Iran; mmsd_22@yahoo.com (S.K.); ahmadi.p@ut.ac.ir (P.A.); 3 Department of Chemistry, Faculty of Science, Tonekabon Branch, Islamic Azad University, Tonekabon 46841-61167, Iran; nabipoor1370@gmail.com; 4 School of Chemistry, College of Science, University of Tehran, Tehran 14179-35840, Iran; s.ghafarimovahed@ut.ac.ir; 5 Department of Mechanical Wood Technology, Faculty of Forest Industry, University of Forestry, 1797 Sofia, Bulgaria; 6 Department of Nanotechnology, Faculty of Engineering Modern Technologies, Amol University of Special Modern Technologies (AUSMT), Amol 46156-64616, Iran; ali.dorieh@yahoo.com; 7 Faculty of Chemical Technologies, University of Chemical Technology and Metallurgy, 1757 Sofia, Bulgaria; stpetrin@uctm.edu; 8 Department of Wood Industry, Faculty of Applied Sciences, Universiti Teknologi MARA Pahang Branch Jengka Campus, Bandar Tun Abdul Razak 26400, Pahang, Malaysia; leesenghua@uitm.edu.my; 9 Institute for Infrastructure Engineering and Sustainable Management (IIESM), Universiti Teknologi MARA, Shah Alam 40450, Selangor, Malaysia

**Keywords:** starch-based adhesive, citric acid, urea, medium-density fiberboard (MDF), formaldehyde-free binder

## Abstract

Formaldehyde-free starch adhesives offer a renewable alternative to conventional binders used in medium-density fiberboard (MDF), but their application remains limited by poor moisture resistance and insufficient mechanical performance. The aim of this research work was to investigate and evaluate the combined effects of sorbitol and glycerol plasticizers, citric acid modification, and urea addition on the properties of starch-based adhesives and the resulting MDF panels. Five adhesive formulations were prepared and characterized using Fourier-transform infrared spectroscopy (FTIR) and differential scanning calorimetry (DSC). Laboratory MDF panels were manufactured and evaluated for thickness swelling (TS), water absorption (WA), internal bond strength (IB), modulus of rupture (MOR), and modulus of elasticity (MOE). Among the tested formulations, the hybrid adhesive system, composed of sorbitol/starch/glycerol/urea/citric acid (SSGUC), provided the best overall performance. Panels bonded with SSGUC exhibited the lowest TS and WA after both 2 and 24 h of water immersion, with TS_2_ values of 22.74 to 24.54% and WA_2_ and WA_24_ values of 91.16–101.43% and 132.11–141.29%, respectively. The same panels achieved IB, MOR, and MOE values of 0.59–0.86 MPa, 24.95–26.17 MPa, and 2413–2632 MPa, respectively. FTIR spectral changes were consistent with citric-acid-mediated esterification and modified hydrogen-bonding interactions in the urea-containing formulations, while DSC revealed formulation-dependent differences in thermal behavior. The FTIR interpretation was based primarily on comparative band development and intensity changes rather than on marginal peak-position shifts. Overall, the results indicate that combining sorbitol and glycerol with citric acid and urea can improve the moisture resistance and mechanical performance of starch-bonded MDF. Further optimization, including rheological characterization and comparison with commercial binders, is required to assess industrial applicability.

## 1. Introduction

Wood adhesives play a vital role in the production of engineered wood products, including plywood, particleboard, oriented strand board (OSB), and medium-density fiberboard (MDF) [1,2]. The steadily increasing global demand for wood-based panels has consequently stimulated the extensive production and use of adhesive systems. Among these products, MDF remains predominantly bonded with synthetic formaldehyde-based resins, particularly urea-formaldehyde (UF), melamine-urea-formaldehyde (MUF), and, to a lesser extent, phenol-formaldehyde (PF). Their widespread industrial application is primarily attributable to their high reactivity, strong bonding performance, short hot-pressing cycles, thermal stability, and cost-effectiveness [3,4,5,6]. Despite their favourable technological performance and economic advantages, the widespread use of UF resins in the wood-based panel industry has become increasingly controversial because these adhesives may release residual and hydrolytically generated formaldehyde during panel manufacture and throughout the service life of the finished products. Formaldehyde is classified by the International Agency for Research on Cancer (IARC) as carcinogenic to humans, while prolonged or elevated exposure has also been associated with irritation of the eyes and respiratory tract, allergic sensitization, asthma-like symptoms, and other adverse health effects [7,8]. These concerns, together with increasingly stringent formaldehyde-emission regulations and growing consumer demand for healthier indoor environments, have intensified efforts to reduce dependence on conventional formaldehyde-based binders. At the same time, the fossil-derived origin and end-of-life implications of many synthetic adhesive systems present additional challenges in terms of resource depletion, carbon emissions, recyclability, and circular material use. Consequently, considerable research attention is being directed toward sustainable, biodegradable, and low-emission adhesives derived from renewable resources [9,10,11,12,13]. The development of high-performance bio-adhesives therefore represents an important pathway toward safer wood products, reduced dependence on petrochemical feedstocks, and closer alignment of the wood-based panel sector with the principles of green chemistry, resource efficiency, and the circular bioeconomy.

Among the renewable biopolymers investigated for wood bonding, starch has emerged as one of the most promising candidates because of its abundance, low cost, biodegradability, broad availability from agricultural resources, and chemically modifiable structure. Starch is a polysaccharide composed primarily of amylose, a predominantly linear α-(1 → 4)-linked glucan, and amylopectin, a highly branched macromolecule containing α-(1 → 4)-linked chains and α-(1 → 6) branch points. Their relative proportions vary according to botanical origin and strongly influence starch gelatinization, viscosity, retrogradation, film formation, and adhesive performance [14,15]. When heated in water, starch granules absorb moisture, swell, lose their native semicrystalline organization, and release polymer chains into the surrounding aqueous phase, producing a viscous, cohesive paste suitable for application to lignocellulosic substrates [16,17]. Adhesion is promoted principally through hydrogen bonding and other secondary interactions between the hydroxyl-rich starch macromolecules and the polar functional groups present on wood fibers, together with mechanical interlocking following adhesive penetration into surface irregularities. Despite these favourable characteristics, native starch has important limitations as a binder for MDF manufacturing. Its numerous accessible hydroxyl groups impart pronounced hydrophilicity, resulting in extensive moisture absorption, dimensional instability, and loss of bond integrity under humid or wet conditions. Native starch adhesives may also exhibit high or unstable viscosity, limited storage stability, retrogradation, brittleness after drying, and insufficient cohesive strength to withstand the demanding conditions experienced during panel manufacture and service [16,17,18,19]. Although the strong affinity of starch for water facilitates gelatinization, initial wet tack, and adhesive distribution, it ultimately compromises the moisture resistance of the cured bond. Consequently, native starch generally cannot meet MDF performance requirements without physical or chemical modification. Several modification routes, including oxidation, etherification, esterification, grafting, and covalent or ionic crosslinking, have therefore been investigated to reduce starch hydrophilicity and reinforce its polymeric network. Among these approaches, crosslinking with multifunctional organic acids, particularly citric acid, is attractive because ester bonds can form between carboxyl groups and starch hydroxyl groups during thermal curing. This reaction can restrict chain mobility, reduce the number of accessible hydroxyl groups, and improve the cohesive strength and water resistance of the resulting adhesive. Aldehyde-based modifiers such as glyoxal and dialdehyde starch have also been explored because of their ability to generate more highly interconnected networks. Nevertheless, increasing crosslink density may reduce molecular mobility and produce a rigid or brittle adhesive layer, creating a persistent trade-off between moisture durability, cohesive strength, and flexibility [20,21,22,23].

Plasticizers are commonly introduced to moderate this effect. Glycerol and sorbitol can disrupt intermolecular starch-starch hydrogen bonding, increase free volume and chain mobility, facilitate film formation, and reduce cracking of the cured adhesive. Their molecular size and hygroscopicity, however, produce different effects on viscosity, flexibility, migration, and moisture sensitivity. Glycerol generally provides efficient plasticization but may increase water uptake, whereas sorbitol can offer greater dimensional stability because of its lower mobility. The type and concentration of plasticizer must therefore be carefully balanced against the degree of chemical crosslinking to avoid either an excessively brittle network or an overly mobile and moisture-sensitive matrix [24,25,26].

Coupling agents and reinforcing components, including silanes and micro- or nanofibrillated cellulose, have also been investigated to enhance interfacial interactions and stress transfer between starch adhesives and wood fibers. More complex starch-based systems have demonstrated the potential of combining complementary modification mechanisms. For example, Neitzel et al. [27] investigated dialdehyde starch adhesives containing emulsifiable polymeric methylene diphenyl diisocyanate and microfibrillated cellulose for MDF production. The resulting panels approached the performance of boards bonded with commercial MUF resin, illustrating the benefits of integrating chemical modification, secondary crosslinking, and fiber reinforcement. Nevertheless, the incorporation of fossil-derived isocyanates reduces the renewable content of such systems and does not provide a completely formaldehyde- and isocyanate-free solution [28,29].

Despite substantial progress, many starch-based adhesive formulations rely on a single crosslinker or plasticizer, although an industrially relevant MDF binder must simultaneously provide suitable viscosity and fiber distribution, storage and processing stability, controlled curing, sufficient flexibility, strong adhesion, and moisture durability. Optimizing one property frequently compromises another, making it unlikely that a single modifier can satisfy the entire performance profile [16,17,20]. For example, citric-acid-mediated crosslinking can restrict starch-chain mobility and improve water resistance, but excessive crosslinking may produce a rigid and brittle network. Conversely, plasticizers improve chain mobility, flexibility, and film formation but may reduce cohesive strength and increase moisture sensitivity at unsuitable concentrations. Premature gelatinization at high solids content, inconsistent curing behaviour, plasticizer migration, and inadequate long-term wet durability further emphasize the need for carefully optimized multicomponent systems [24,25].

Although sorbitol and glycerol have been individually investigated as starch plasticizers, their combined effects in citric-acid-modified systems containing urea remain insufficiently understood. In such formulations, sorbitol and glycerol may exert complementary plasticizing effects, citric acid may promote thermally induced esterification, and urea may modify hydrogen-bonding interactions within the starch matrix and at the adhesive–wood interface. Combining these mechanisms could provide a better compromise between processability, flexibility, network cohesion, and moisture resistance than formulations based on individual modifiers. The combined use of sorbitol and glycerol therefore offers a potentially useful route for balancing flexibility, cohesion, and water resistance in starch-based adhesive systems.

In addition, urea may influence hydrogen-bond organization and network development during adhesive preparation and hot pressing. Nevertheless, the combined effects of dual plasticization, citric acid modification, and urea incorporation on starch-based adhesive performance in MDF manufacture remain insufficiently understood. Accordingly, the aim of this study was to determine how sorbitol/glycerol plasticization, citric acid modification, and urea addition affect adhesive structure, thermal behavior, and MDF panel performance. It was hypothesized that the formulation containing both plasticizers together with citric acid and urea would provide the most favorable balance between moisture resistance and mechanical performance.

## 2. Material and Methods

### 2.1. Materials

Food-grade corn starch (moisture content: 11–12 wt%; Sigma-Aldrich, Darmstadt, Germany) was used as the primary polymeric substrate for adhesive preparation. Sorbitol (≥99%, Merck, Darmstadt, Germany) and glycerol (≥99.5%, Merck, Germany) were employed as plasticizers. Citric acid monohydrate (≥99%, Sigma-Aldrich, Germany) served as the primary crosslinking agent, while urea (analytical grade, ≥99%, Merck, Germany) was used as a secondary modifier in hybrid adhesive systems. Deionized water (electrical conductivity < 1 μS cm^−1^) was used throughout all experiments. All chemicals were used as received without further purification. Wood fibers used for laboratory MDF panel manufacture were obtained from a commercial MDF producer (Kimia Company, Gorgan, Iran) and consisted predominantly of mixed hardwood fibers. Before blending, the fibers were dried to an initial moisture content of 5 ± 0.5 wt% and were conditioned to ensure consistent furnish preparation.

### 2.2. Adhesive Formulations

Five starch-based adhesive formulations were prepared to investigate the effects of plasticizer type and the addition of urea to citric-acid-modified starch systems. Each formulation was adjusted to a final wet batch mass of 1000 g. The component masses and the nominal water balance for each formulation are given in Table 1. After completion of the modification stage, the adhesive was cooled to room temperature (23 ± 1 °C) and stored in sealed containers prior to characterization and MDF panel fabrication. The composition, functional role of each component, and identification code of the investigated formulations are presented in Table 1.

### 2.3. Adhesive Preparation

A standardized multistage procedure was used for all formulations. Starch was first dispersed in water and gelatinized, after which the plasticizer system was incorporated. The pH was then adjusted using an aqueous citric acid solution, and citric acid and, where applicable, urea were introduced under controlled thermal and mixing conditions. The total batch mass was finally adjusted to 1000 g, and the adhesive was cooled to room temperature before use or storage. This procedure produced homogeneous modified starch adhesives, while further thermally induced network formation was expected to occur during hot pressing of the MDF panels.

### 2.4. Starch Gelatinization

For each adhesive batch, 200 g of corn starch was gradually dispersed in 500 g of deionized water under continuous mechanical agitation at 400–500 rpm using an overhead stirrer (IKA RW20, IKA-Werke GmbH & Co. KG, Staufen, Germany). The resulting suspension was heated gradually to 75–80 °C and maintained within this temperature range for 15 min under continuous stirring to promote starch gelatinization. The transition from an opaque granular suspension to a homogeneous, translucent, and highly viscous paste was used as a visual indication of gelatinization, reflecting granule swelling, disruption of the native starch structure, and the release of polymer chains, particularly amylose, into the aqueous phase.

### 2.5. Plasticizer Incorporation

Following gelatinization, the plasticizer(s) specified for each formulation in Table 1 were gradually added to the hot starch paste under continuous agitation. The mixture was stirred for an additional 10 min while the gelatinization temperature was maintained to promote dissolution and homogeneous distribution of the polyols throughout the starch matrix.

### 2.6. pH Adjustment

Prior to crosslinker addition, the pH of each adhesive formulation was adjusted to 3.5–4.5 using an aqueous citric acid solution (10 wt%). The mass of citric acid introduced during pH adjustment was included in the predetermined total citric acid content of the corresponding formulation. The resulting acidic conditions were intended to support subsequent thermally induced esterification between the carboxyl groups of citric acid and the hydroxyl groups of starch during adhesive preparation and MDF hot pressing.

### 2.7. Crosslinking and Hybrid Modification

Citric acid was incorporated into all formulations at 10 g per batch, corresponding to 5 wt% based on the dry mass of starch. This amount included the citric acid previously introduced during pH adjustment. In the hybrid formulations (SSUC and SSGUC), 20 g of urea, equivalent to 10 wt% based on dry starch, was subsequently added as a secondary modifier. The mixtures were heated to 85 °C and maintained at this temperature for 30 min under continuous stirring to ensure homogeneous distribution of the modifiers and promote intermolecular interactions within the starch matrix. Under these aqueous conditions, the treatment was expected primarily to facilitate hydrogen-bond reorganization and prepare the system for more extensive thermally induced esterification during subsequent hot pressing of the MDF panels.

### 2.8. Final Adjustment and Cooling

After completion of the crosslinking stage, additional deionized water was added to adjust the total batch mass to 1000 g. The adhesive was then cooled to room temperature (23 ± 1 °C) and stored in sealed containers until use. Before application to the fibers, the adhesive systems used for panel manufacture were adjusted to a solids content of approximately 40 wt%, and this adjustment refers to the application stage rather than to every intermediate stage of adhesive synthesis.

### 2.9. Physicochemical Characterization

#### 2.9.1. Fourier Transform Infrared Spectroscopy (FTIR)

The chemical structures and functional-group interactions of the starch-based adhesive formulations were investigated using a Nicolet 6700 Fourier-transform infrared spectrometer (ThermoFisher Scientific, Waltham, MA, USA). Before analysis, representative adhesive samples were dried, ground into a fine powder, and thoroughly mixed with spectroscopic-grade potassium bromide (KBr). The resulting mixtures were compressed into transparent pellets. Spectra were recorded in transmittance mode over the wavenumber range of 4000–400 cm^−1^ at a spectral resolution of 16 cm^−1^, with 200 scans averaged for each sample. A background spectrum of a blank KBr pellet was collected under the same conditions and subtracted from the sample spectra. The spectra were examined for formulation-dependent changes in the O–H, C–H, carbonyl, and C–O–C regions that could indicate altered hydrogen-bonding interactions and thermally induced esterification.

#### 2.9.2. Differential Scanning Calorimetry (DSC)

The thermal behaviour of the starch-based adhesive formulations was examined using a NETZSCH DSC 214 instrument (NETZSCH-Gerätebau GmbH, Selb, Germany) under a nitrogen atmosphere. Approximately 10 mg of each homogenized adhesive sample was placed in an aluminium pan and heated from 20 to 200 °C at a constant rate of 10 °C min^−1^. The DSC analysis was performed to investigate thermal transitions and curing behavior of the modified starch adhesive systems.

#### 2.9.3. Adhesive Synthesis Parameters

The principal processing parameters applied during adhesive synthesis are summarized in Table 2.

These processing conditions were selected based on starch gelatinization kinetics, esterification behavior of citric acid, and the thermal stability of urea.

#### 2.9.4. MDF Panel Fabrication and Property Evaluation

Before panel manufacture, the solids content of each starch-based adhesive was adjusted to approximately 40 wt%. The adhesive was applied to the wood fibers at a dry-solids loading of 10 wt%, based on the oven-dry mass of the fibers to ensure uniform distribution. The adhesive-treated fibers were formed into mats and hot-pressed into laboratory MDF panels with nominal dimensions of 500 mm × 350 mm × 10 mm. Hot-pressing was conducted at 180 °C under a maximum specific pressure of 1.3 MPa for 7 min. The hot-pressing parameters were selected based on preliminary experiments and the requirements of the modified starch system to achieve sufficient bond formation and dimensional consolidation while limiting thermal degradation of the starch matrix and wood fibers. A total of three independently manufactured panels were produced for each adhesive formulation.

Following pressing, the panels were cooled, trimmed, and conditioned at 25 ± 2 °C and 65 ± 5% relative humidity for two weeks before specimen preparation and testing. The target panel density was 700 kg·m^−3^. This value represents the design density used during panel manufacture; where density-related interpretation is provided in the Results and Discussion, it refers only to measured panel density rather than the nominal target value.

The wood fibers used for panel production were obtained from Kimia Company and consisted predominantly of mixed hardwood fibers. Before blending, the fibers were dried to an initial moisture content of 5 ± 0.5 wt%. The furnish was conditioned before mat formation, and adhesive blending was carried out under constant laboratory conditions using a fixed solids-based adhesive loading, a standardized mixing sequence, and the same mixing duration for all formulations in order to improve distribution uniformity. Hot pressing was conducted under fixed platen temperature and pressing time conditions. Although process settings were kept constant, the internal temperature profile of the mat was not directly instrumented in the present study.

Panel density was determined in accordance with EN 323 [30] and reported separately for each adhesive formulation.

Bending strength (MOR) and modulus of elasticity (MOE) were determined in accordance with EN 310 [31]. Internal bond strength (IB), corresponding to tensile strength perpendicular to the panel plane, was evaluated according to EN 319 [32]. Thickness swelling (TS) was determined after 2 and 24 h of water immersion using specimens prepared and tested according to EN 317 [33]. Water absorption (WA) was evaluated using the same specimens and calculated as the percentage increase in mass relative to the initial conditioned mass. Mechanical testing was conducted using an Instron 1400 universal testing machine (Instron, Norwood, MA, USA) equipped with an appropriate load cell.

For each formulation, one specimen was obtained from each of three independently manufactured panels and tested for each property, resulting in three independent observations per formulation and test.

### 2.10. Statistical Analysis

Statistical analysis was performed using SPSS software (Version 26, IBM Corp. and Armonk, NY, USA). A one-way analysis of variance (ANOVA) was conducted to evaluate the effect of adhesive formulation on the measured properties of MDF panels. Mean comparisons were performed using Duncan’s multiple range test at a significance level of *p* < 0.05. All measurements were carried out in triplicate.

## 3. Results and Discussion

### 3.1. Adhesive Characterization

#### 3.1.1. FTIR Analysis

**Figure 1:**
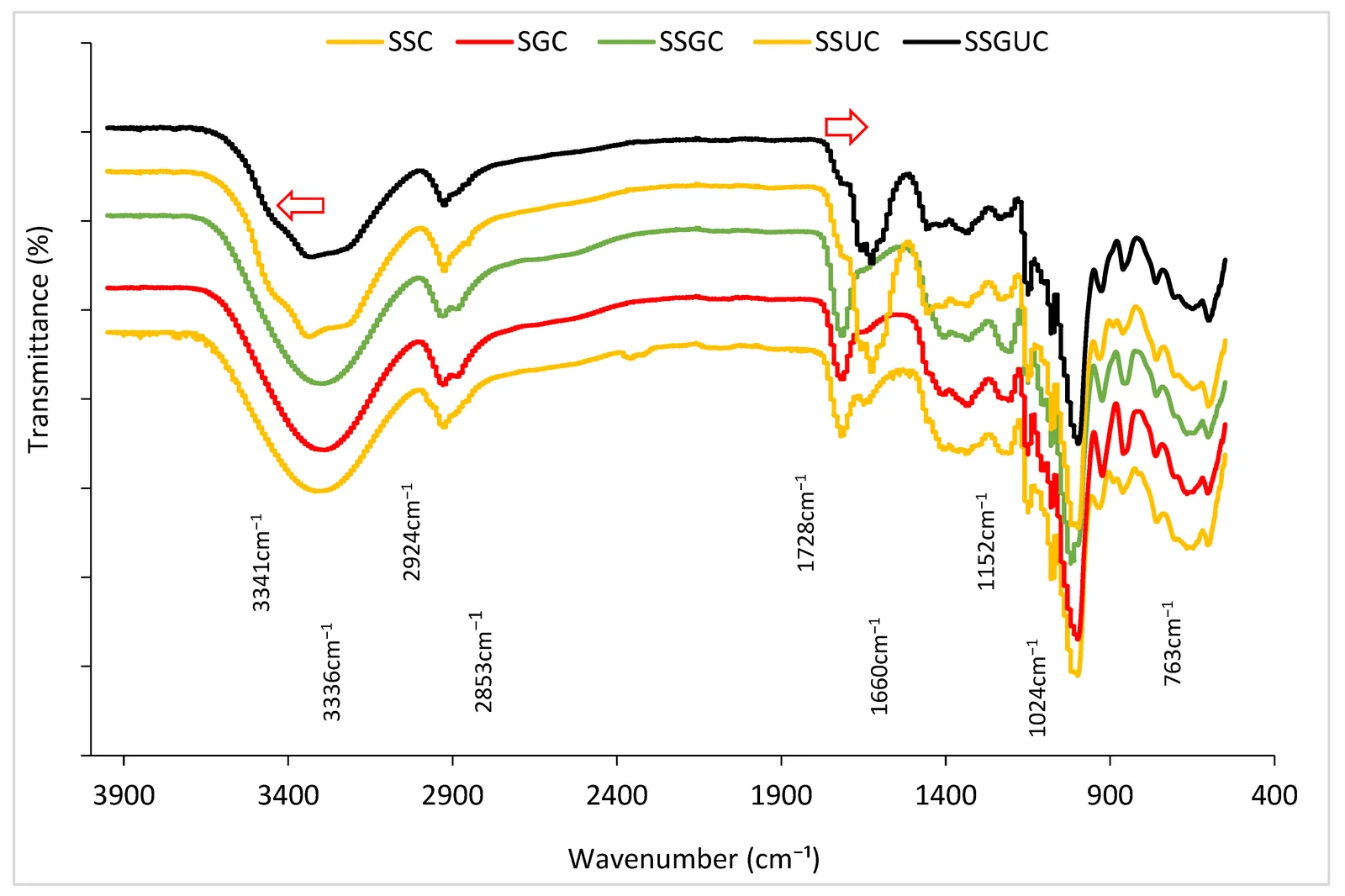
FTIR spectra of the starch-based adhesive systems, showing characteristic changes in O–H, C–H, and carbonyl regions associated with plasticization and citric-acid crosslinking. Red arrows highlight formulation-dependent changes in the O–H and carbonyl regions.

The FTIR spectra of the five adhesive formulations (SSC, SGC, SSGC, SSUC, and SSGUC) exhibit several pronounced peak shifts and intensity changes that collectively confirm the formation of ester and amide linkages and the development of a highly crosslinked starch-based network (Figure 1). All samples retain the characteristic O-H stretching envelope near 3328–3342 cm^−1^; however, the intensity of this band decreases markedly in SSGC, SSUC, and most significantly in SSGUC, accompanied by a subtle blue shift from 3336 cm^−1^ (SSC) to 3341 cm^−1^ (SSGUC). This attenuation and shift reflect the progressive consumption of hydroxyl groups via esterification and partial amide formation, indicating reduced hydrogen bonding and restricted mobility of the starch chains, consistent with trends reported for citric-acid-modified polysaccharides [20]. In the C-H stretching region, peaks at 2924 cm^−1^ and 2853 cm^−1^ become more pronounced in SGC and SSGC due to the addition of glycerol. However, these same peaks show a slight decline in SSGUC, signaling reduced vibrational freedom of alkyl-containing moieties after formation of a denser covalent network. This phenomenon aligns with the structural confinement observed in heavily crosslinked starch plasticizer systems [34].

In the carbonyl region, all formulations contained citric acid, but a more distinct absorption band attributable to esterified and/or strongly hydrogen-bonded carbonyl environments was observed more clearly in SSGC, SSUC, and especially SSGUC. This does not indicate the absence of citric acid in SSC or SGC; rather, it suggests that the extent of esterification and/or the spectroscopic visibility of the corresponding carbonyl environment depended on formulation composition and the local hydrogen-bonding environment. The 1728 cm^−1^ position is fully consistent with esterified starch reported in Carbohydrate Polymers [35,36]. In SSGUC, the sharpness and amplitude of the 1728 cm^−1^ peak provide strong evidence for extensive esterification and higher crosslink density. In the hybrid formulations SSUC and SSGUC, an additional band in the amide-related region near 1658–1662 cm^−1^ was observed, consistent with the presence of urea-associated interactions and possible reaction-derived structures formed during thermal treatment [37].

Changes in the fingerprint region, particularly near 1152 and 1024 cm^−1^, were also more evident in the hybrid and dual-plasticized systems, supporting the view that these formulations underwent greater structural reorganization. Overall, the FTIR results support the conclusion that the SSGUC system experienced the most pronounced formulation-dependent structural modification among the tested adhesives, which is consistent with its superior MDF performance. However, these assignments are interpreted as supportive qualitative evidence rather than definitive proof based on marginal band displacements alone.

#### 3.1.2. Differential Scanning Calorimetry (DSC) Analysis

**Figure 2:**
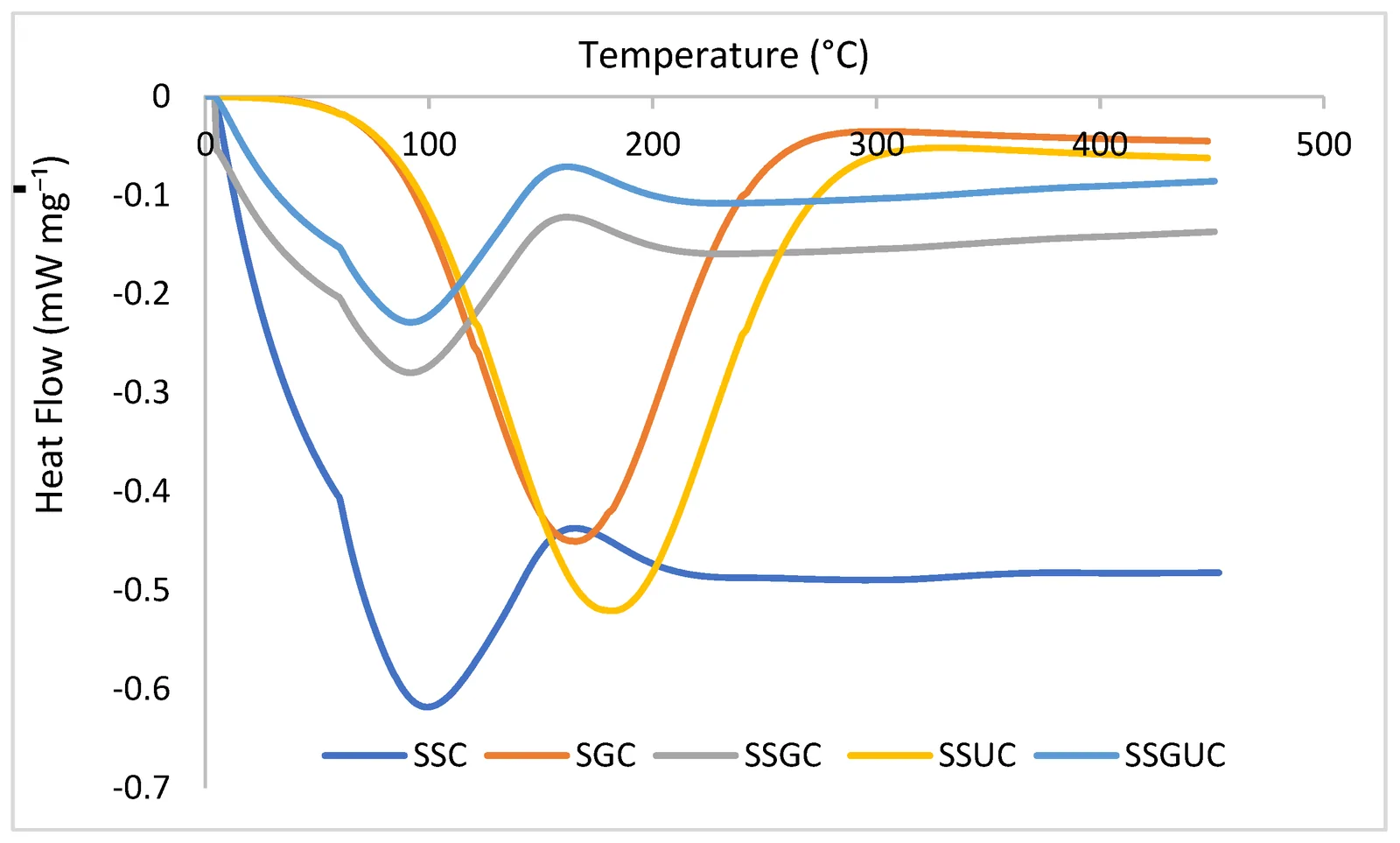
DSC thermograms of the starch-based adhesive formulations, illustrating the influence of plasticizer type and urea modification on thermal transitions.

The DSC thermograms of the five starch-based adhesive formulations are presented in Figure 2. All formulations exhibited broad endothermic transitions over approximately 50–200 °C, although both the position and intensity of the thermal events varied considerably among the different formulations. Such broad endothermic regions are commonly attributed to the evaporation of free and bound water together with molecular relaxation within the amorphous starch matrix, where extensive hydrogen-bonding interactions govern the thermal response [25,38]. The differences observed among the formulations therefore suggest that the incorporation of different plasticizers and modifiers altered the intermolecular interactions within the adhesive network. The SSC formulation showed a pronounced endothermic peak at approximately 95–105 °C, whereas the corresponding transition in SGC shifted to a higher temperature (around 160–170 °C). This difference indicates that replacing sorbitol with glycerol modified the thermal behavior of the starch matrix. Because sorbitol and glycerol differ in molecular structure and plasticizing efficiency, they influence hydrogen bonding, water retention, and chain mobility in different ways, leading to distinct thermal responses during heating [24,25]. Among the hybrid formulations, SSUC exhibited a broad endothermic transition centred at approximately 180–190 °C, followed by a gradual recovery toward the baseline. Compared with SSC, this higher-temperature transition suggests stronger intermolecular interactions within the modified starch system. The combined presence of citric acid and urea may contribute to this behavior through changes in hydrogen-bonding interactions and the formation of thermally induced ester linkages during curing. Similar effects of citric-acid modification on the thermal characteristics of starch-based materials have been reported previously [20,22]. For the urea-containing systems, especially SSGUC, the thermal response became broader and more distributed over the upper part of the scanned temperature range, indicating a more heterogeneous thermal behavior. These broad events are not attributed solely to water evaporation or relaxation phenomena. They may also include overlapping formulation-dependent processes such as thermally induced reorganization, onset of acid-catalyzed bond cleavage, and early-stage degradation-related events, particularly in citric-acid-modified systems.

This broader response suggests a wider distribution of molecular environments within the adhesive matrix, which may result from the simultaneous incorporation of sorbitol, glycerol, citric acid, and urea. The combination of two plasticizers is expected to modify chain mobility while promoting a more homogeneous distribution of intermolecular interactions, leading to a broader thermal transition rather than a single well-defined endothermic event [24,25]. It should be noted that DSC alone cannot verify crosslinking density or thermal degradation resistance. Instead, the thermograms primarily reflect differences in molecular mobility, water–polymer interactions, and thermal transitions among the formulations. Nevertheless, the broader thermal response observed for SSGUC is consistent with the superior physical and mechanical performance of the corresponding MDF panels, suggesting that the combined modification strategy produced a more stable adhesive network than the individual formulations.

### 3.2. Thickness Swelling (TS_2_ and TS_24_)

Thickness swelling (TS) is a key parameter of the dimensional stability and moisture durability of wood-based panels. It reflects the combined effects of water penetration into the porous fiber network, hygroscopic expansion of the wood cell walls, and moisture-induced weakening or disruption of adhesive bonds. This property is particularly important for panels bonded with starch-based adhesives because starch contains numerous accessible hydroxyl groups that readily interact with water through hydrogen bonding. Water uptake can plasticize the starch matrix, increase polymer-chain mobility, and reduce adhesive cohesion, while swelling of the wood fibers further generates internal stresses at the adhesive–fiber interface [14,16,17]. Reducing the accessibility of hydrophilic groups and improving the integrity of the cured adhesive network are therefore important for limiting moisture penetration and preserving the dimensional stability of starch-bonded MDF.

**Figure 3:**
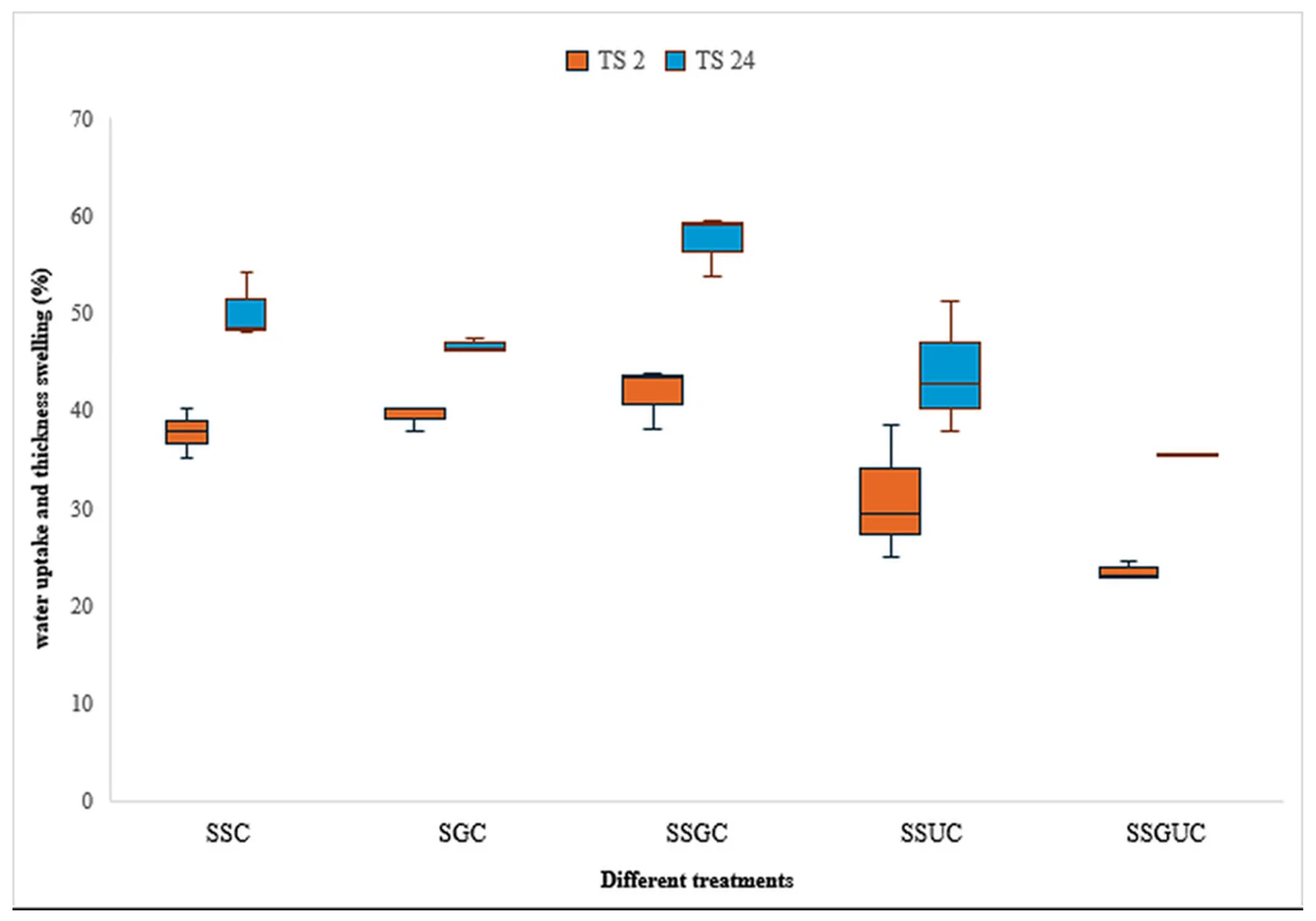
Thickness swelling after 2- and 24 h immersion of the laboratory starch-bonded MDF panels.

As illustrated in Figure 3, the TS_2_ values differed significantly among the adhesive formulations, although the response did not follow a uniform decreasing trend across the entire formulation series. Panels bonded with SSC exhibited TS_2_ values of 35.18–40.10%, indicating substantial dimensional changes during the initial 2 h of immersion. This behaviour can be attributed to the hydrophilic character of the starch-rich matrix and to the limited resistance of the adhesive network to rapid water penetration. Although citric acid can promote thermally induced esterification with hydroxyl groups in starch and wood during hot pressing, the resulting modification at the applied concentration may not have been sufficient to prevent water from reaching the remaining accessible polar sites. In addition, residual unreacted citric acid and sorbitol may contribute to the hydrophilicity of the cured adhesive phase. Once water enters the panel, plasticization of the starch network, expansion of the wood fibers, and interfacial stresses can occur simultaneously, resulting in the observed thickness increase [20,39]. The relatively broad TS_2_ range recorded for SSC also indicates variability among the independently manufactured panels. Such variation may arise from differences in actual panel density, vertical density profile, adhesive distribution, local fiber compaction, or moisture release during hot pressing.

The panels bonded with SGC exhibited TS_2_ values of 37.96–40.27%, which were comparable to those obtained for SSC (35.18–40.10%). Thus, under the investigated conditions, replacing sorbitol with glycerol did not produce an evident improvement in short-term dimensional stability. This response may be related to the greater plasticizing efficiency and hygroscopicity of glycerol. By disrupting intermolecular hydrogen bonding among starch chains, glycerol increases chain mobility and improves the flexibility of the adhesive film; however, its small molecular size, high affinity for water, and ability to increase free volume within the polymer matrix may also facilitate moisture diffusion [14,26]. Consequently, any benefit associated with improved adhesive distribution and reduced brittleness may have been offset by the increased moisture sensitivity of the glycerol-plasticized network.

The dual-plasticizer SSGC formulation produced TS_2_ values of 38.01–43.81%, representing the highest upper range among the three urea-free systems. The combined incorporation of sorbitol and glycerol may have provided greater chain mobility and film flexibility but did not improve resistance to short-term water exposure. Instead, the two hydrophilic polyols may have increased the number of water-accessible sites and the free volume available for moisture transport, particularly if they were not fully incorporated into the thermally modified starch network. These results suggest that dual plasticization alone was insufficient to counteract the inherent hydrophilicity of starch and that improved flexibility does not necessarily translate into enhanced dimensional stability.

In contrast to the urea-free formulations, the urea-containing SSUC and SSGUC systems generally provided better short-term dimensional stability. The most pronounced improvement was observed for SSGUC, which exhibited the lowest and narrowest TS_2_ range of 22.74–24.54%. Relative to the minimum and maximum values recorded for SSC, this corresponds to an approximate reduction of 35–39% in thickness swelling. The limited variation among the SSGUC panels also suggests more consistent performance under short-term water exposure. The improved behaviour of the urea-containing formulations may be associated with complementary interactions among citric acid, urea, starch, and wood fibers. During hot pressing, citric acid can promote esterification with hydroxyl groups in starch and lignocellulosic constituents, while urea can participate in extensive hydrogen-bonding interactions with the surrounding hydroxyl-rich matrix [40,41]. These interactions may increase network cohesion, improve adhesive–fiber contact, and reduce the mobility of starch chains during water exposure. Urea may also influence the distribution and thermal response of citric acid within the formulation. However, because the present analyses do not directly quantify crosslink density or distinguish newly formed covalent bonds from physical interactions, the mechanism should be regarded as a plausible interpretation rather than definitive evidence of synergistic crosslinking.

After 24 h of immersion, the differences among the formulations became more pronounced. The SSC and SGC panels exhibited TS_24_ values within the overall range of 46.02–54.28%, confirming that the corresponding sorbitol- and glycerol-plasticized systems remained highly susceptible to prolonged moisture exposure. The SSGC formulation showed TS_24_ values of 53.75–59.41% and therefore presented the greatest long-term swelling among the investigated systems. This result indicates that combining sorbitol and glycerol without urea did not improve long-term dimensional stability and may have increased moisture sensitivity through the combined hygroscopicity and plasticizing effects of the two polyols.

The response of SSUC was more variable, with TS_24_ values ranging from 37.93 to 51.31%. Although its lower values indicate an improvement relative to the urea-free formulations, the broad range suggests that the protective effect was not equally developed in all panels. Variations in adhesive distribution, panel density, moisture removal, or the extent of thermally induced network formation may have contributed to this variability. By comparison, SSGUC consistently exhibited the lowest TS_24_ values, remaining close to 35%. Its superior performance suggests that the combination of sorbitol and glycerol became beneficial when used together with citric acid and urea, possibly because the plasticizers maintained sufficient flexibility while the citric-acid–urea modification enhanced cohesion and resistance to moisture-induced deformation.

### 3.3. Water Absorption (WA_2_ and WA_24_)

**Figure 4:**
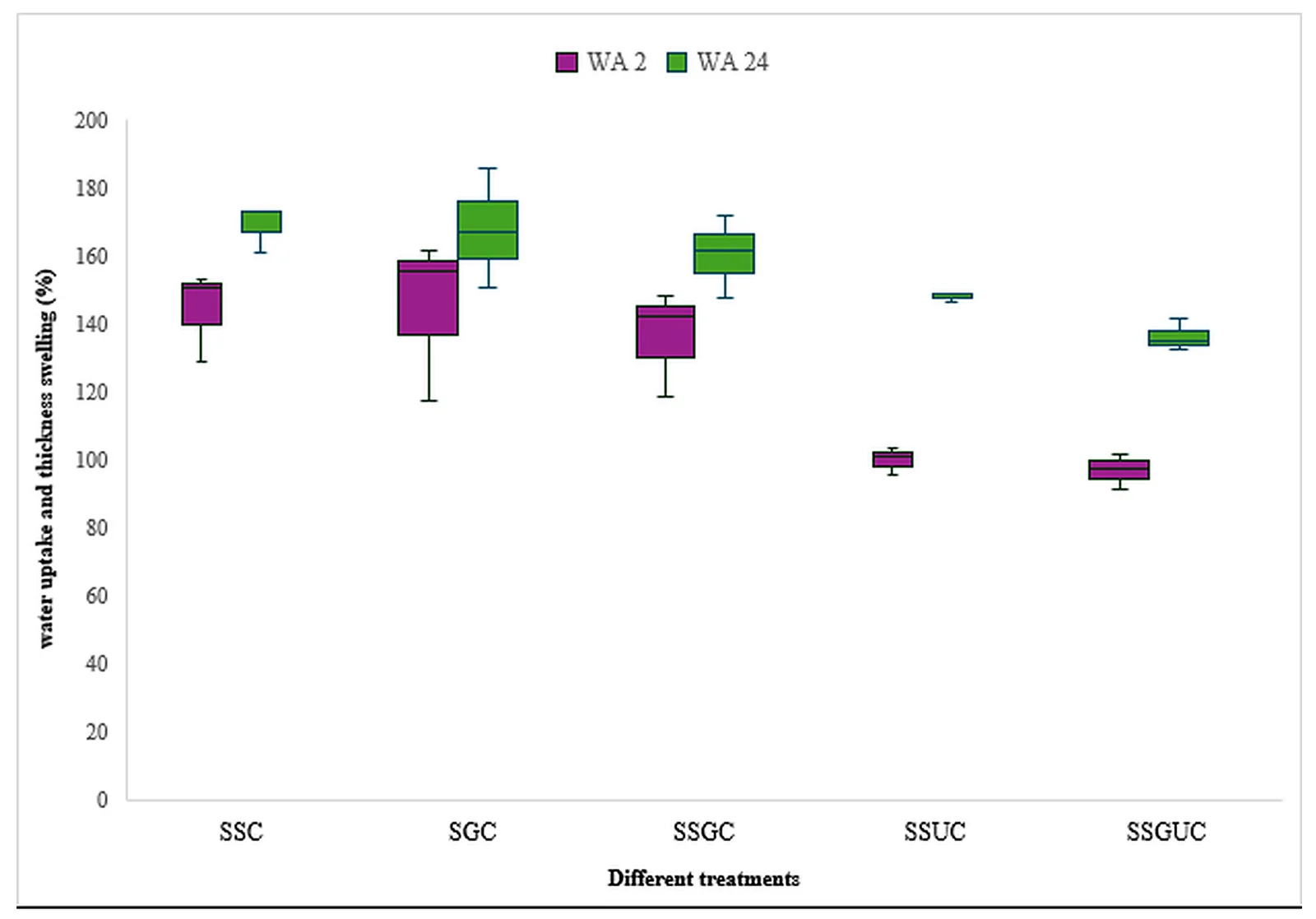
Water absorption after 2- and 24 h immersion of the laboratory starch-bonded MDF panels.

Water absorption (WA) is closely related to thickness swelling and provides additional insight into the interaction between moisture and the adhesive fiber network. High WA values are typically associated with the hydrophilic nature of starch-based adhesives and the porous structure of lignocellulosic materials [16,42]. As shown in Figure 4, water absorption after 2 h of immersion (WA_2_) varied considerably among the adhesive formulations. Panels bonded with SSC and SGC exhibited relatively high WA_2_ values (117.50–161.55%), indicating rapid water penetration into the board structure. This behaviour can be attributed to the abundance of accessible hydroxyl groups in starch molecules, which strongly attract water molecules through hydrogen bonding [14]. Similar observations have been reported for other starch-based wood adhesives, where insufficient crosslinking results in high moisture sensitivity [41,43]. The SSGC formulation showed moderately reduced water absorption (118.53–148.18%). The presence of both sorbitol and glycerol may improve the flexibility of the adhesive network and promote better interfacial contact between fibers and adhesive; however, the plasticizing effect of these polyols may also increase free volume within the polymer matrix, facilitating water diffusion [26]. In contrast, the urea-modified systems exhibited significantly lower WA_2_ values. The SSUC formulation ranged from 95.41 to 103.37%, while SSGUC showed the lowest water absorption (91.16–101.43%). The reduced water uptake observed in these systems suggests that urea contributes to strengthening intermolecular interactions within the adhesive network. Previous studies have shown that nitrogen-containing compounds can enhance hydrogen-bond interactions and improve the cohesion of starch-based adhesive matrices, thereby reducing water penetration [42,44].

After 24 h of immersion (WA_24_), the same trend remained evident. SSC and SGC exhibited the highest water absorption values (160.90–185.54%), confirming the relatively low water resistance of these systems. SSGC showed intermediate absorption (147.77–171.64%), while SSUC and SSGUC again demonstrated superior resistance to moisture. In particular, SSGUC consistently exhibited the lowest WA_24_ values (132.11–141.29%). The improved performance of the SSGUC system indicates that the combined action of dual plasticizers and hybrid crosslinking agents enhances the structural stability of the adhesive matrix and limits water diffusion into the panel structure. Similar improvements in water resistance have been reported for modified starch adhesives where crosslinking reactions reduce the availability of hydrophilic hydroxyl groups and increase network compactness [39,44]. The reduced water uptake in SSGUC is consistent with the restricted molecular mobility observed in DSC thermograms, suggesting a denser network that limits the penetration of water molecules into the starch matrix. Overall, the WA results are consistent with the thickness swelling data and further confirm that the sorbitol/glycerol–citric acid/urea hybrid system provides the most effective strategy for improving the moisture resistance of starch-based MDF adhesives.

### 3.4. Internal Bond Strength (IB)

**Figure 5:**
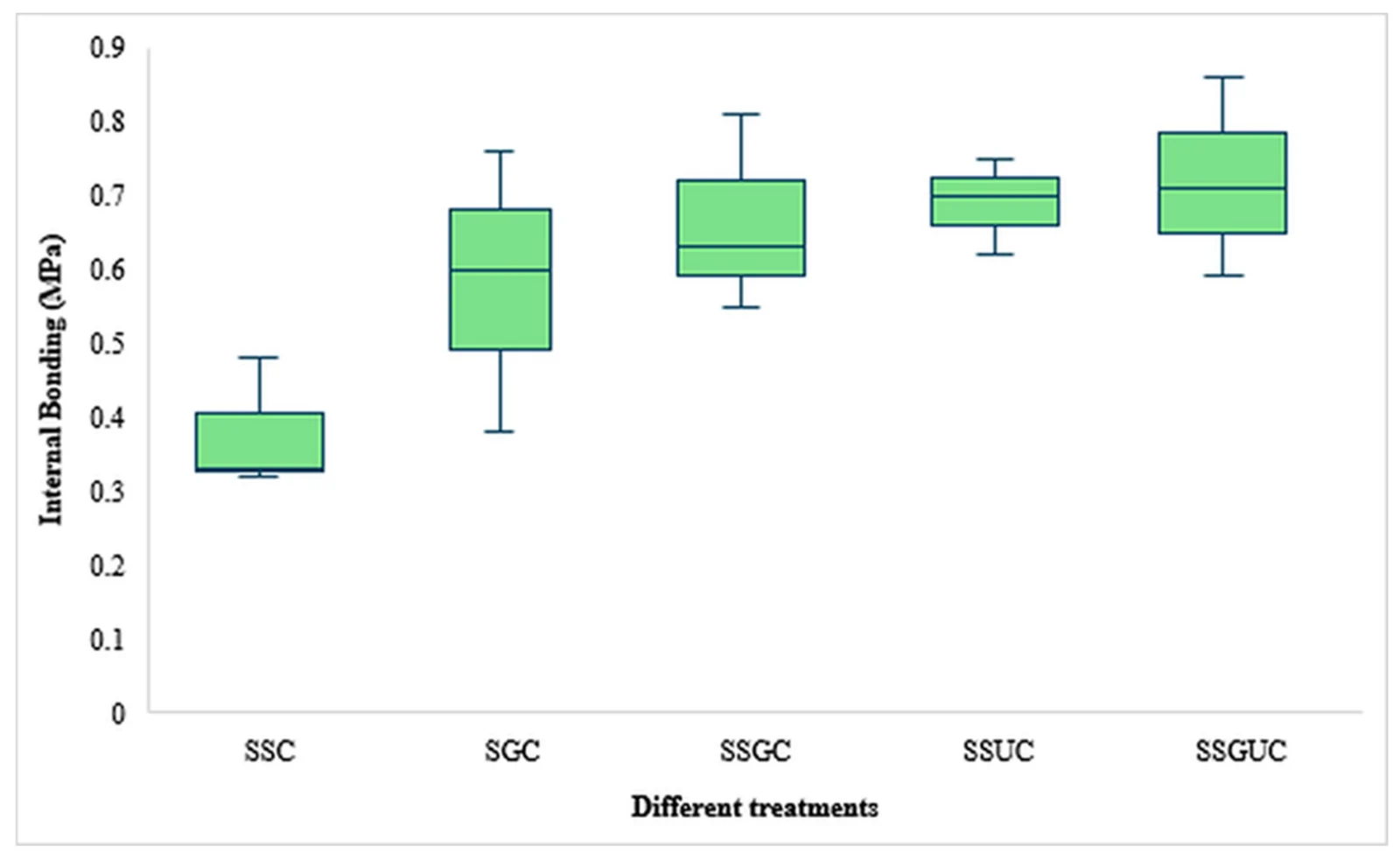
Internal bond strength of the laboratory starch-bonded MDF panels.

Internal bond strength (IB) reflects the tensile strength perpendicular to the panel surface and is strongly affected by adhesive cohesion, crosslink density, and the fiber-adhesive interface [16]. As illustrated in Figure 5, significant differences were observed among the five adhesive formulations. Panels bonded with SSC showed the lowest IB values (0.32–0.48 MPa). This limited bonding performance agrees with earlier findings on native or lightly modified starch adhesives, where excessive hydroxyl groups and insufficient crosslinking create a weak and moisture-sensitive network [41,44]. The higher TS and WA values measured earlier further support this interpretation. The SGC formulation showed more variable IB strength (0.38–0.76 MPa), likely due to the plasticization effect of glycerol. Although glycerol enhances flexibility and improves stress transfer between fibers, excessive chain mobility can reduce cohesive strength in some regions [26].

Clear improvements appeared in SSGC, SSUC, and SSGUC, confirming the expected synergistic effects of plasticizers and crosslinking agents. SSGC reached IB values of 0.55–0.81 MPa, suggesting better fiber penetration and internal cohesion. The urea-modified adhesives outperformed all other systems. SSUC (0.62–0.75 MPa) and particularly SSGUC (0.59–0.86 MPa) achieved the highest bonding performance among the formulations. This improvement can be attributed to two interacting mechanisms: (a) citric acid forms ester bridges with starch hydroxyl groups, producing a 3D crosslinked network; (b) urea enhances intermolecular hydrogen bonding among starch chains and wood fibers. This improvement in mechanical strength is directly supported by the FTIR results, where the intense ester carbonyl peak at 1700 cm^−1^ indicates a high degree of covalent crosslinking.

Such dual interaction increases network density, restricts polymer mobility, and improves moisture resistance consistent with earlier literature [39,45]. The highest IB value (0.86 MPa) falls within the range reported for optimized modified starch adhesives (0.4–0.9 MPa) and meets typical MDF standards, highlighting the practical applicability of SSGUC.

### 3.5. Modulus of Rupture (MOR)

**Figure 6:**
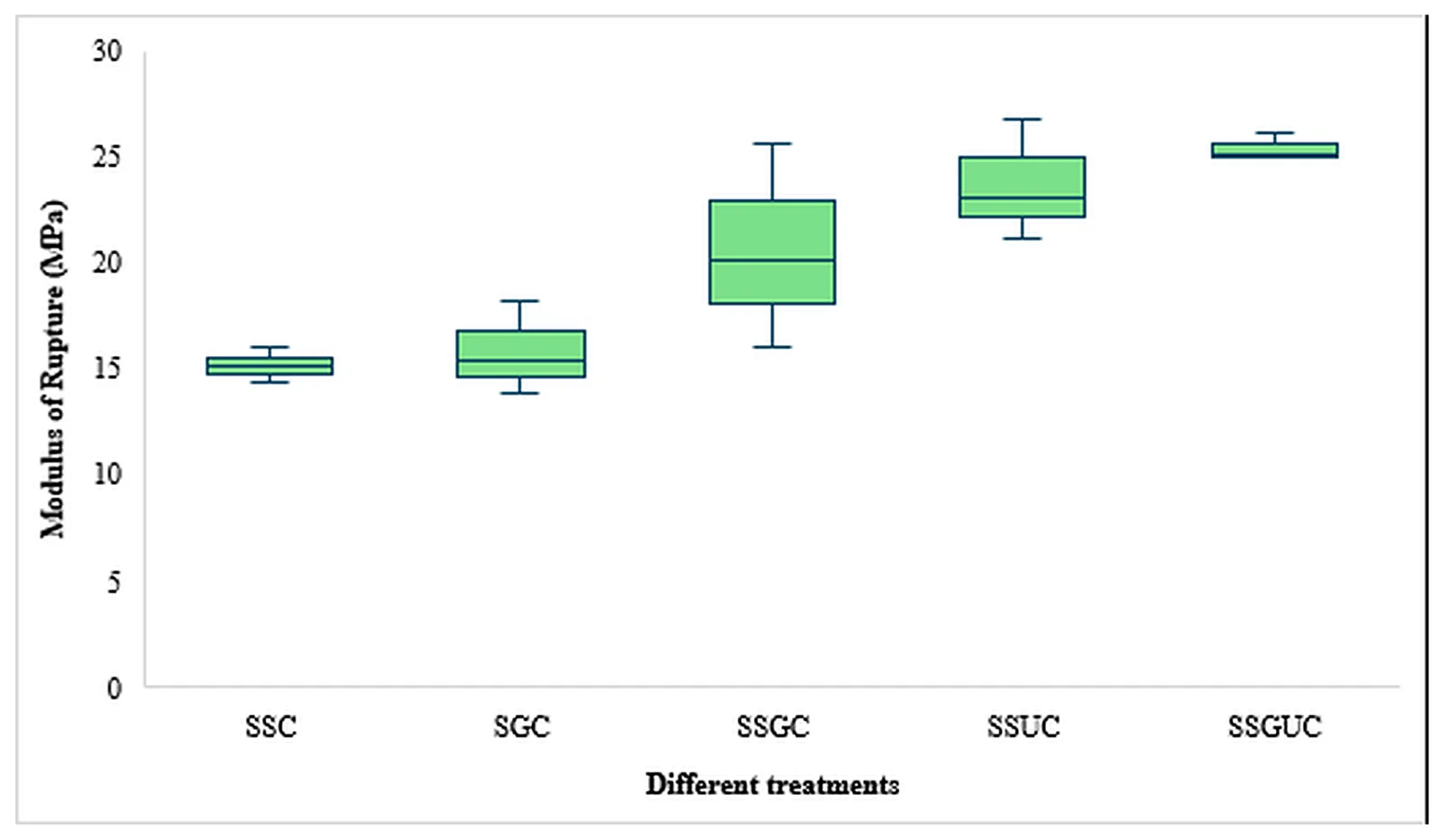
Modulus of rupture of the laboratory starch-bonded MDF panels.

The modulus of rupture (MOR) represents the maximum bending stress sustained by a panel before failure and is influenced by adhesive cohesion, fiber-adhesive interfacial bonding, fiber distribution, panel density, and the vertical density profile. As shown in Figure 6, the SSC and SGC panels exhibited the lowest MOR values, ranging from 13.91 to 18.16 MPa. These results indicate that using either sorbitol or glycerol as the sole plasticizer did not provide sufficient adhesive-network integrity to ensure consistently high bending strength. The relatively poor dimensional stability of these panels further suggests that their adhesive networks remained susceptible to moisture-induced weakening, although the dry MOR response cannot be attributed exclusively to water sensitivity or crosslink density.

The SSGC formulation produced a broader and generally higher MOR range of 16.01–25.68 MPa. This improvement suggests that the combined use of sorbitol and glycerol provided a more favourable balance between adhesive flexibility and cohesive integrity than either plasticizer used individually. Plasticizers can reduce brittleness by increasing starch-chain mobility, allowing the cured adhesive layer to accommodate local deformation and transfer stress more effectively between adjacent fibers [26]. Nevertheless, the wide range observed for SSGC indicates appreciable variation among the independently manufactured panels, potentially arising from differences in adhesive distribution, panel density, mat consolidation, or the local development of the fiber network.

The urea-containing formulations showed the highest overall bending performance. SSUC achieved MOR values of 21.12–26.84 MPa, while SSGUC exhibited a narrower range of 24.95–26.17 MPa. Although the highest individual MOR value was recorded for SSUC, SSGUC provided the highest lower-bound value and the most consistent bending strength among the investigated formulations. Relative to the lowest value measured for SSC and SGC, the minimum MOR of SSGUC was approximately 79% higher. This response indicates that incorporating urea into the citric-acid-modified adhesive systems improved the capacity of the panels to resist bending loads.

The enhanced MOR of SSUC and SSGUC may be associated with complementary effects within the adhesive network. Thermally induced esterification involving citric acid can increase cohesion and reduce excessive chain mobility, whereas urea may modify hydrogen-bonding interactions among starch molecules and between the adhesive and wood fibers. In SSGUC, the simultaneous presence of sorbitol and glycerol may additionally provide sufficient flexibility to reduce brittle failure while maintaining effective stress transfer across the fiber network. Similar improvements in the bending strength of wood composites have been associated with chemical modification of starch and reinforcement through covalent and secondary interactions [44,45].

### 3.6. Modulus of Elasticity (MOE)

**Figure 7:**
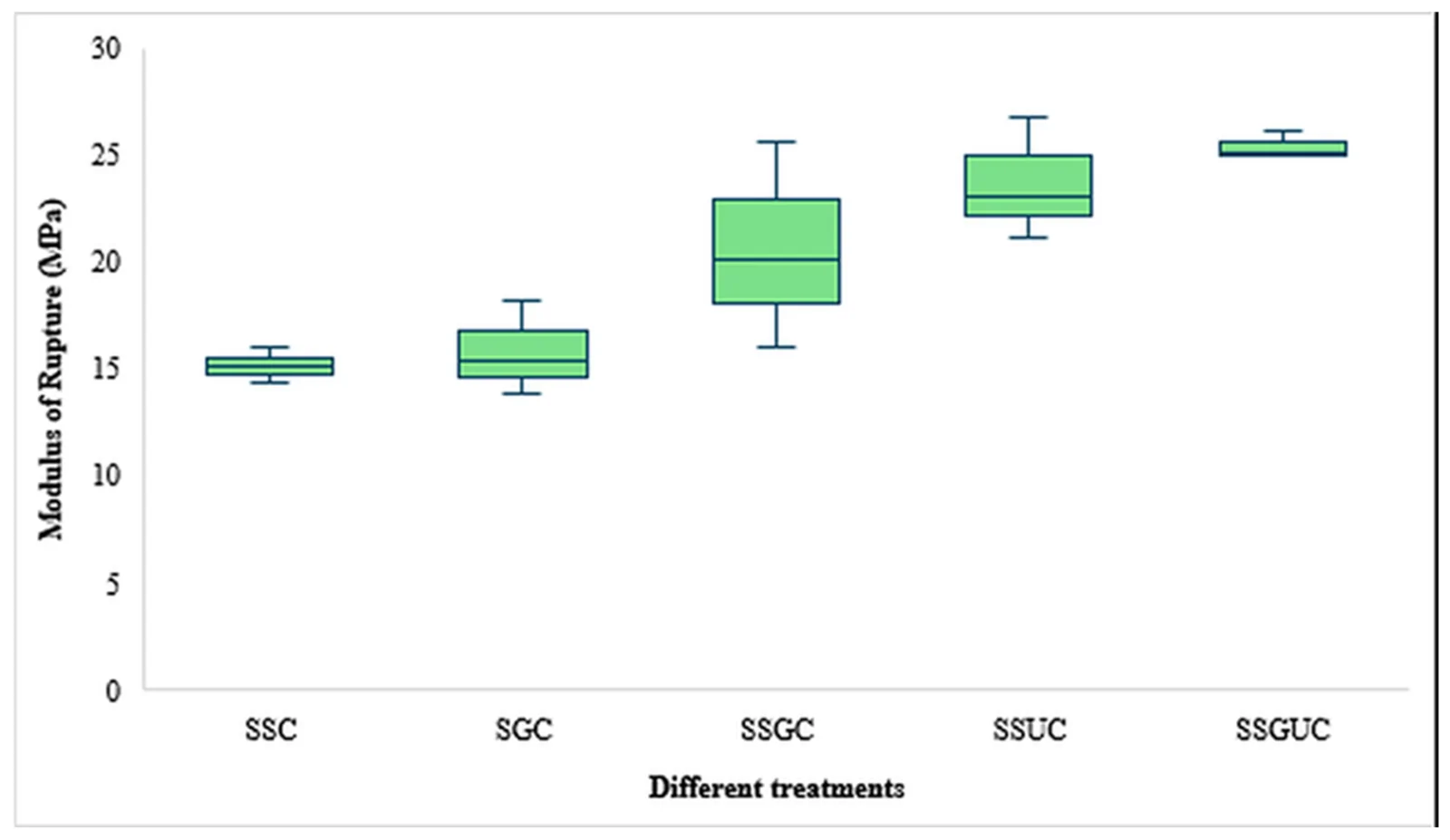
Modulus of elasticity of the laboratory starch-bonded MDF panels.

The modulus of elasticity (MOE) characterizes panel stiffness within the elastic deformation range and reflects the efficiency with which stresses are transferred through the consolidated fiber network. In addition to adhesive performance, MOE is strongly influenced by fiber geometry and orientation, panel density, the vertical density profile, and mat consolidation. As shown in Figure 7, SSC exhibited the lowest and most variable MOE values, ranging from 819 to 2205 MPa. This broad range suggests inconsistent stiffness among the independently manufactured panels and may indicate variations in adhesive distribution, fiber consolidation, or panel density. Although the result is compatible with a relatively weakly integrated starch-citric acid matrix, the variation precludes attributing the response solely to adhesive composition.

The SGC and SSGC panels exhibited moderately higher MOE values within an overall range of 1801–2531 MPa. Although glycerol and sorbitol generally increase molecular mobility and may soften the starch phase, suitable plasticization can improve adhesive spreading, reduce local brittleness, and promote more uniform bonding among fibers. These effects may facilitate stress transfer through the panel structure and thereby increase macroscopic stiffness, provided that the plasticizer content does not excessively reduce adhesive cohesion. The narrower MOE ranges obtained for these formulations relative to SSC also suggest greater uniformity in panel performance.

The urea-containing systems exhibited the highest overall stiffness. SSUC produced MOE values of 2215–2623 MPa, while SSGUC showed values of 2413–2632 MPa. Although the maximum values of SSUC and SSGUC were similar, SSGUC exhibited the highest minimum value and the narrowest performance range, indicating consistently high stiffness. Relative to the lowest MOE recorded for SSC, the minimum value obtained for SSGUC was nearly three times higher. This improvement may reflect stronger adhesive cohesion, more effective fiber-to-fiber stress transfer, and a more uniformly consolidated panel structure.

The enhanced MOE of the urea-containing panels may be associated with complementary interactions among starch, citric acid, urea, the plasticizers, and wood fibers. Thermally induced citric-acid esterification may restrict excessive starch-chain mobility, whereas urea may reorganize hydrogen-bonding interactions within the adhesive matrix and at the fiber interface. In SSGUC, dual plasticization may additionally reduce brittle discontinuities without causing an excessive loss of network integrity. Comparable increases in stiffness have been reported for chemically modified starch systems in which improved cohesion and interfacial bonding enhanced stress transfer within lignocellulosic composites [38,39].

## 4. Conclusions

This study demonstrated that combining sorbitol and glycerol plasticization with citric acid modification and urea addition can improve the performance of starch-based adhesives for MDF production. Among the five formulations investigated, SSGUC provided the most favourable overall balance between moisture resistance and mechanical performance. Panels bonded with this formulation exhibited the lowest thickness swelling and water absorption after both 2 and 24 h of immersion, together with consistently high internal bond strength, modulus of rupture, and modulus of elasticity. These findings indicate that the combined modification strategy was more effective than using either plasticizer individually or dual plasticization without urea.

FTIR spectral changes were consistent with thermally induced citric acid esterification and altered hydrogen-bonding interactions within the modified starch matrix, while DSC revealed formulation-dependent differences in thermal behaviour. However, these techniques provide indirect evidence and do not independently confirm crosslink density or the formation of specific amide structures. The improved performance of SSGUC may therefore be attributed to complementary effects of citric acid, urea, and the two plasticizers on adhesive cohesion, flexibility, and fiber-adhesive interactions.

From a practical perspective, the SSGUC system represents a promising formaldehyde-free starch-based binder prepared from readily available components and compatible with conventional laboratory MDF hot pressing. Nevertheless, the remaining moisture sensitivity, particularly after 24 h of immersion, indicates that further formulation optimization is required before application under humid or moisture-demanding service conditions. Although the present results demonstrate that dual plasticization combined with citric acid and urea can improve MDF panel performance, several aspects require further investigation before industrial implementation. In particular, future work should include rheological characterization of the adhesive systems (viscosity, thixotropy, and flow behavior), direct thermal mass-loss analysis under inert and oxidizing atmospheres, and comparison with commercial MDF binders under industrially relevant process conditions.

## Figures and Tables

**Table 1:** Formulations of starch-based adhesive systems.

Code	Starch	Sorbitol	Glycerol	Citric Acid	Urea	Deionized Water (g)
SSC	200 g	15 g	0 g	10 g	0 g	775
SGC	200 g	0 g	20 g	10 g	0 g	770
SSGC	200 g	7.5 g	10 g	10 g	0 g	772.5
SSUC	200 g	10 g	0 g	10 g	20 g	760
SSGUC	200 g	7.5 g	7.5	10 g	20 g	755

Note: The deionized-water values are nominal balances calculated as 1000 g minus the masses of the listed ingredients, with starch weighed as received. They include the water used to prepare the 10 wt% citric acid solution. After heating, deionized water was added as needed to restore the final batch mass to 1000 g. The values describe the final formulation and do not represent the cumulative amount of water added during preparation, which may be higher because of evaporation.

**Table 2:** Principal processing parameters for adhesive synthesis.

Stirring speed	400–500 rpm
Gelatinization temperature	75–80 °C
Crosslinking temperature	85 °C
Crosslinking pH	3.5–4.5
Cooling temperature	23 ± 1 °C
Total batch mass	1000 g

## Data Availability

The original contributions presented in this study are included in the article. Further inquiries can be directed to the corresponding authors.

## References

[1] Mantanis G.I., Athanassiadou E.T., Barbu M.C., Wijnendaele K. (2018). Adhesive systems used in the European particleboard, MDF and OSB industries. Wood Mater. Sci. Eng..

[2] Tobisch S., Dunky M., Hänsel A., Krug D., Wenderdel C., Niemz P., Teischinger A., Sandberg D. (2023). Survey of Wood-Based Materials. Springer Handbook of Wood Science and Technology.

[3] Dorieh A., Ayrilmis N., Farajollah Pour M., Ghafari Movahed S., Valizadeh Kiamahalleh M., Shahavi M.H., Hatefnia H., Mehdinia M. (2022). Phenol formaldehyde resin modified by cellulose and lignin nanomaterials: Review and recent progress. Int. J. Biol. Macromol..

[4] Dorieh A., Farajollah Pour M., Ghafari Movahed S., Pizzi A., Pouresmaeel Selakjani P., Valizadeh Kiamahalleh M., Hatefnia H., Shahavi M.H., Aghaei R. (2022). A review of recent progress in melamine-formaldehyde resin based nanocomposites as coating materials. Prog. Org. Coat..

[5] Dorieh A., Pouresmaeel Selakjani P., Shahavi M.H., Pizzi A., Ghafari Movahed S., Farajollah Pour M., Aghaei R. (2022). Recent developments in the performance of micro/nanoparticle-modified urea-formaldehyde resins used as wood-based composite binders: A review. Int. J. Adhes. Adhes..

[6] Dorieh A., Pahlavan F., Hájková K., Hýsek Š., Farajollah Pour M., Fini E.H. (2024). Advancing Sustainable Building Materials: Reducing Formaldehyde Emissions in Medium Density Fiber Boards with Lignin Nanoparticles. Adv. Sustain. Syst..

[7] Khoshakhlagh A.H., Mohammadzadeh M., Sicard P., Bamel U. (2024). Human exposure to formaldehyde and health risk assessment: A 46-year systematic literature review. Environ. Geochem. Health.

[8] La Torre G., Vitello T., Cocchiara R.A., Della Rocca C. (2023). Relationship between formaldehyde exposure, respiratory irritant effects and cancers: A review of reviews. Public Health.

[9] Ghafari Movahed S., Rezvani I., Dorieh A., Kamrani S., Mehdinia M., Pourpilehkesh M., Shahavi M.H., Nabipoor S., Antov P., Savov V. (2026). Recent Advances and Sustainability Perspectives of Biobased Wood Panel Adhesives: Toward Cleaner and Formaldehyde-Free Wood Products. Polymers.

[10] Antov P., Savov V., Neykov N. (2020). Sustainable bio-based adhesives for eco-friendly wood composites. A review. Wood Res..

[11] Gonçalves D., Bordado J.M., Marques A.C., Galhano dos Santos R. (2021). Non-Formaldehyde, Bio-Based Adhesives for Use in Wood-Based Panel Manufacturing Industry—A Review. Polymers.

[12] Hemmilä V., Adamopoulos S., Karlsson O., Kumar A. (2017). Development of sustainable bio-adhesives for engineered wood panels—A review. RSC Adv..

[13] Ferdosian F., Pan Z., Gao G., Zhao B. (2017). Bio-Based Adhesives and Evaluation for Wood Composites Application. Polymers.

[14] Tester R.F., Karkalas J., Qi X. (2004). Starch—Composition, fine structure and architecture. J. Cereal Sci..

[15] Din Z.U., Chen L., Xiong H., Wang Z., Ullah I., Lei W., Shi D., Alam M., Ullah H., Khan S.A. (2020). Starch: An Undisputed Potential Candidate and Sustainable Resource for the Development of Wood Adhesive. Starch/Stärke.

[16] Watcharakitti J., Win E.E., Nimnuan J., Smith S.M. (2022). Modified Starch-Based Adhesives: A Review. Polymers.

[17] Maulana M.I., Lubis M.A.R., Febrianto F., Lee S.H., Iswanto A.H., Antov P., Kristak L., Mardawati E., Sari R.K., Zaini L.H. (2022). Environmentally Friendly Starch-Based Adhesives for Bonding High-Performance Wood Composites: A Review. Forests.

[18] Xu Q., Wen J., Wang Z. (2016). Preparation and Properties of Cassava Starch-Based Wood Adhesives. BioResources.

[19] Tondi G., Wieland S., Wimmer T., Schnabel T., Petutschnigg A. (2012). Starch-sugar synergy in wood adhesion science: Basic studies and particleboard production. Eur. J. Wood Wood Prod..

[20] Reddy N., Yang Y. (2010). Citric acid cross-linking of starch films. Food Chem..

[21] González Seligra P., Medina Jaramillo C., Famá L., Goyanes S. (2016). Biodegradable and non-retrogradable eco-films based on starch–glycerol with citric acid as crosslinking agent. Carbohydr. Polym..

[22] Shi R., Bi J., Zhang Z., Zhu A., Chen D., Zhou X., Zhang L., Tian W. (2007). Characterization of citric acid/glycerol co-plasticized thermoplastic starch prepared by melt blending. Carbohydr. Polym..

[23] Wang N., Yu J., Chang P.R., Ma X. (2007). Influence of Citric Acid on the Properties of Glycerol-Plasticized Dry Starch and Dry Starch/Poly(lactic acid) Blends. Starch/Stärke.

[24] Ma X., Yu J., He K., Wang N. (2007). The Effects of Different Plasticizers on the Properties of Thermoplastic Starch as Solid Polymer Electrolytes. Macromol. Mater. Eng..

[25] Zullo R., Iannace S. (2009). The effects of different starch sources and plasticizers on film blowing of thermoplastic starch: Correlation among process, elongational properties and macromolecular structure. Carbohydr. Polym..

[26] Avérous L., Boquillon N. (2004). Biocomposites based on plasticized starch: Thermal and mechanical behaviours. Carbohydr. Polym..

[27] Neitzel N., Hosseinpourpia R., Adamopoulos S. (2023). A Dialdehyde Starch-Based Adhesive for Medium-Density Fiberboards. BioResources.

[28] Lubis M.A.R., Park B.-D., Hong M.-K. (2020). Tuning of adhesion and disintegration of oxidized starch adhesives for the recycling of medium density fiberboard. BioResources.

[29] Lubis M.A.R., Park B.-D., Hong M.-K. (2019). Tailoring of oxidized starch’s adhesion using crosslinker and adhesion promotor for the recycling of fiberboards. J. Appl. Polym. Sci..

[30] (1993). Wood-Based Panels—Determination of Density.

[31] (1993). Wood-Based Panels—Determination of Modulus of Elasticity in Bending and of Bending Strength.

[32] (1993). Particleboards and Fibreboards—Determination of Tensile Strength Perpendicular to the Plane of the Board.

[33] (1993). Particleboards and Fibreboards—Determination of Swelling in Thickness After Immersion in Water.

[34] Delval F., Crini G., Bertini S., Morin-Crini N., Badot P.-M., Vebrel J., Torri G. (2004). Characterization of crosslinked starch materials with spectroscopic techniques. J. Appl. Polym. Sci..

[35] Yadav P., Sabith M., Bosco S.J.D. (2023). Effect of Citric Acid Modification on Physio-Chemical, Structural, Rheological, Thermal Properties, and In Vitro Digestibility of Amaranthus paniculatus (Rajgeera) Starch. Starch/Stärke.

[36] Mei J.-Q., Zhou D.-N., Jin Z.-Y., Xu X.-M., Chen H.-Q. (2015). Effects of citric acid esterification on digestibility, structural and physicochemical properties of cassava starch. Food Chem..

[37] de Zea Bermudez V., Carlos L.D., Alcácer L. (1999). Sol–Gel Derived Urea Cross-Linked Organically Modified Silicates. 1. Room Temperature Mid-Infrared Spectra. Chem. Mater..

[38] Masina N.P., Choonara Y.E., Kumar P., du Toit L.C., Govender M., Indermun S., Pillay V. (2017). A review of the chemical modification techniques of starch. Carbohydr. Polym..

[39] Wang Y., Xiong H., Wang Z., Zia-ud-Din, Chen L. (2017). Effects of different durations of acid hydrolysis on the properties of starch-based wood adhesive. Int. J. Biol. Macromol..

[40] Chen X., Pizzi A., Zhang B., Zhou X., Fredon E., Gérardin C., Du G. (2022). Particleboard bio-adhesive by glyoxalated lignin and oxidized dialdehyde starch crosslinked by urea. Wood Sci. Technol..

[41] Imam S.H., Mao L., Chen L., Greene R.V. (1999). Wood Adhesive from Crosslinked Poly(Vinyl Alcohol) and Partially Gelatinized Starch: Preparation and Properties. Starch/Stärke.

[42] Lee S.H., Md Tahir P., Lum W.C., Tan L.P., Bawon P., Park B.-D., Osman Al Edrus S.S., Abdullah U.H. (2020). A Review on Citric Acid as a Green Modifying Agent and Binder for Wood. Polymers.

[43] Wang Z., Li Z., Gu Z., Hong Y., Cheng L. (2012). Preparation, characterization and properties of starch-based wood adhesive. Carbohydr. Polym..

[44] Gu Y., Cheng L., Gu Z., Hong Y., Li Z., Li C. (2019). Preparation, characterization and properties of starch-based adhesive for wood-based panels. Int. J. Biol. Macromol..

[45] Chen L., Wang Y., Zia-ud-Din, Fei P., Jin W., Xiong H., Wang Z. (2017). Enhancing the performance of starch-based wood adhesive by silane coupling agent (KH570). Int. J. Biol. Macromol..

